# NK cell heparanase controls tumor invasion and immune surveillance

**DOI:** 10.1172/JCI183295

**Published:** 2024-07-01

**Authors:** Eva M. Putz, Alyce J. Mayfosh, Kevin Kos, Deborah S. Barkauskas, Kyohei Nakamura, Liam Town, Katharine J. Goodall, Dean Y. Yee, Ivan K.H. Poon, Nikola Baschuk, Fernando Souza-Fonseca-Guimaraes, Mark D. Hulett, Mark J. Smyth

Original citation: *J Clin Invest*. 2017;127(7):2777–2788. https://doi.org/10.1172/JCI92958

Citation for this retraction: *J Clin Invest*. 2024;134(13):e183295. https://doi.org/10.1172/JCI183295

QIMR Berghofer Medical Research Institute recently notified the *JCI* of concerns regarding Figure 3C and Supplemental Figure 4A and indicated that an independent panel investigation concluded that these figures are based on data fabricated by Mark J. Smyth. In addition, the investigative panel concluded that Figures 3D and 3E are based on unreliable or unconfirmed data produced by Mark J. Smyth. The QIMR Berghofer Medical Research Institute investigation found that one author, Mark J. Smyth, was the sole individual responsible for provision of data in Figure 3, C–E, and Supplemental Figure 4A. Due to these data integrity concerns, the *JCI* is retracting this article. No issues were raised with regard to any of the other data in the article.

Kevin Kos has agreed with the *JCI*’s decision to retract the paper. The remaining authors abstained from commenting or could not be reached.

## Version 1 07/01/2024

Electronic publication

